# Molecular epidemiology and immunopathogenesis of bovine viral diarrhea virus: a growing threat to regional cattle industry of Xinjiang, China

**DOI:** 10.3389/fmicb.2025.1617998

**Published:** 2025-07-14

**Authors:** Jindong Gao, Lei Kuang, Jinhua Yin, Mengdi Zhang, Md. F. Kulyar, Changmin Hu

**Affiliations:** ^1^College of Animal Science and Technology, Tarim University, Alar, China; ^2^Key Laboratory of Tarim Animal Husbandry Science and Technology, Xinjiang Production and Construction Corps, Alar, China; ^3^College of Veterinary Medicine, Huazhong Agricultural University, Wuhan, China

**Keywords:** bovine viral diarrhea, bovine viral diarrhea virus, prevalence, epidemiology, pathogenesis, Xinjiang

## Abstract

Bovine viral diarrheal virus (BVDV), identified as a serious global pathogen, presents a significant and formidable challenge for the global cattle sector. This presents not only serious economic consequences but also serious immunopathological consequences causing lasting impacts. In the Chinese province of Xinjiang, where the ruminants are high and the topographical features unique and contribute towards complex epidemiology, multiple genotypes of the BVDV are continuously interacting and adapting. This includes the persistent presence of the earlier predominant BVDV1, the rising trend towards the antigenically complex BVDV2, and the recent presence of the HoBi-like (BVDV3) strain, contributing toward complexity. The purpose of this review is to present a detailed analysis relative to the molecular epidemiology, genomic diversity levels, and complex pathology associated with the persistence and spreading of the infection by the BVDV. In light of the country’s inability to execute the efficacious eradication of the infection, stringent biosafety measures need to be adopted, the genomic investigation has to be increased, and the establishment of the multi-valent vaccine for the induction of the local strain of the infection should be encouraged. These steps need to be taken towards the rising danger of the presence of the BVDV, not only towards the province but also towards the larger territories beyond.

## 1 Introduction

Bovine viral diarrhea (BVD) is considered a widespread infection in cattle accompanied by diarrhea, high fever, necrosis and mucosal erosion of the gastrointestinal tract, leukopenia, and reduced platelets (Peddireddi et al., 2018). BVD is a viral disease caused by bovine viral diarrhea virus (BVDV), which is the enveloped/positively stranded RNA virus and belonging to the genus Pestivirus and the Flaviviridae family. BVDV has three genotypes: BVDV1 (Pestivirus A), BVDV2 (Pestivirus B), and BVDV3 (Pestivirus H); the latter one is also termed a HoBi-like genotype. Cytopathic and non-cytopathic are its two biotypes, which are classified depending upon their impact on cultured cells; the former induces apoptosis (Pedrera et al., 2009; Ran et al., 2019; Wang et al., 2021). BVDV might be sub-clinical, mild, severe, and/or fatal, severely affects dairy industry and infects yaks, buffaloes, goats, pigs, sheep, and deer, resulting in huge economic losses for the cattle industry around the globe, particularly in China (Fulton et al., 2002; Ran et al., 2019; Wang et al., 2021). BVDV causes an average annual production loss of €42.14 to €67.19 per animal (Pinior et al., 2019). In addition, instead of the major symptom of diarrhea, BVDV-infected animals are often susceptible to respiratory infections, reproductive issues, growth retardation, stillbirth, and even mucosal diseases, leading to immunosuppression and disruption of an innate immune response (Tautz et al., 2015). Based on the phylogenetic analysis and genetic characterization, the epidemiological and molecular features of BVDV were investigated, which revealed that BVDV1 and BVDV1b are the most prevalent subtypes of BVDV in China (Wang et al., 2020). Further, in Beijing, the highest prevalence was reported because 93.4% of animals were positive for BVDV antibodies (Weng et al., 2015). The positive rate of BVDV in dairy animals in Fujian, Shaanxi, and Shandong is 90, 88.9, and 83.3%, respectively (Ran et al., 2019). Likewise, in Xinjiang, a 53.68% positive rate of BVDV antibodies is reported (Yang et al., 2023). Xinjiang Uygur Autonomous Region is located in north-western China and has an established animal-husbandry industry consisting of around 4 million cattle in its main districts, including Kuitun, Shawan, Yili, Manas, Aksu, Hami, and Shihezi. As it borders with Kazakhstan, Central Asian countries, and Russia, a higher risk of cross-border BVDV transmission is predicted. Thus, this review aims to assess the genetic characterization, prevalence, epidemiological patterns, pathogenic mechanisms, immunosuppression, and preventive measures for BVDV in Xinjiang compared with other Chinese cities/provinces.

## 2 Xinjiang might be at a higher risk of BVDV infection

Xinjiang has a larger (57 million hectares) natural grassland area that is naturally cultivatable with fertile land, supporting a diverse group of livestock rearing. Due to its geographical location, Xinjiang is considered among the main pastoral areas of China and is ranked second in contributing cattle production proportion, resulting in rapid progress in developing animal husbandry practices. Thus, most farmers prefer intensive and large-scale animal production. However, due to the continuously expanding breeding scale in Xinjiang, different epidemics have emerged, and BVDV is seriously affecting cattle production at a large scale, posing a continuous risk of BVDV infection for healthy animals (Yang et al., 2023). Additionally, as Xinjiang borders Kazakhstan, Central Asian countries, and Russia and is an immediate neighboring region of China, it is anticipated a higher risk of cross-border BVDV transmission. Although no direct evidence has revealed that Xinjiang’s BVDV prevalence is attributed to its close borders with Asian countries, due to its geographical location and continuous developments in the rapid breeding practices of cattle, it might contribute to the spread of infection. Moreover, the alternative reason why Xinjiang is at higher risk of BVDV infection rate is more convincing. Zhong et al. (2011) reported that BVDV1c has been found circulating in Xinjiang, with higher similarities (94.4%) with the Australian strains. Most of the Holstein cattle in Xinjiang were often imported from Australia, and as BVDV1c is reported to be a predominant subtype of BVDV in Australia, the healthy herd of cattle is also at higher risk, which may lead to increased levels of persistent infection. Therefore, finding a strategy that helps reduce the BVDV risk rate in Xinjiang is an urgent requirement.

## 3 Genetic characterization and prevalence of BVDV in Xinjiang and other Chinese provinces

**Figure 1:**
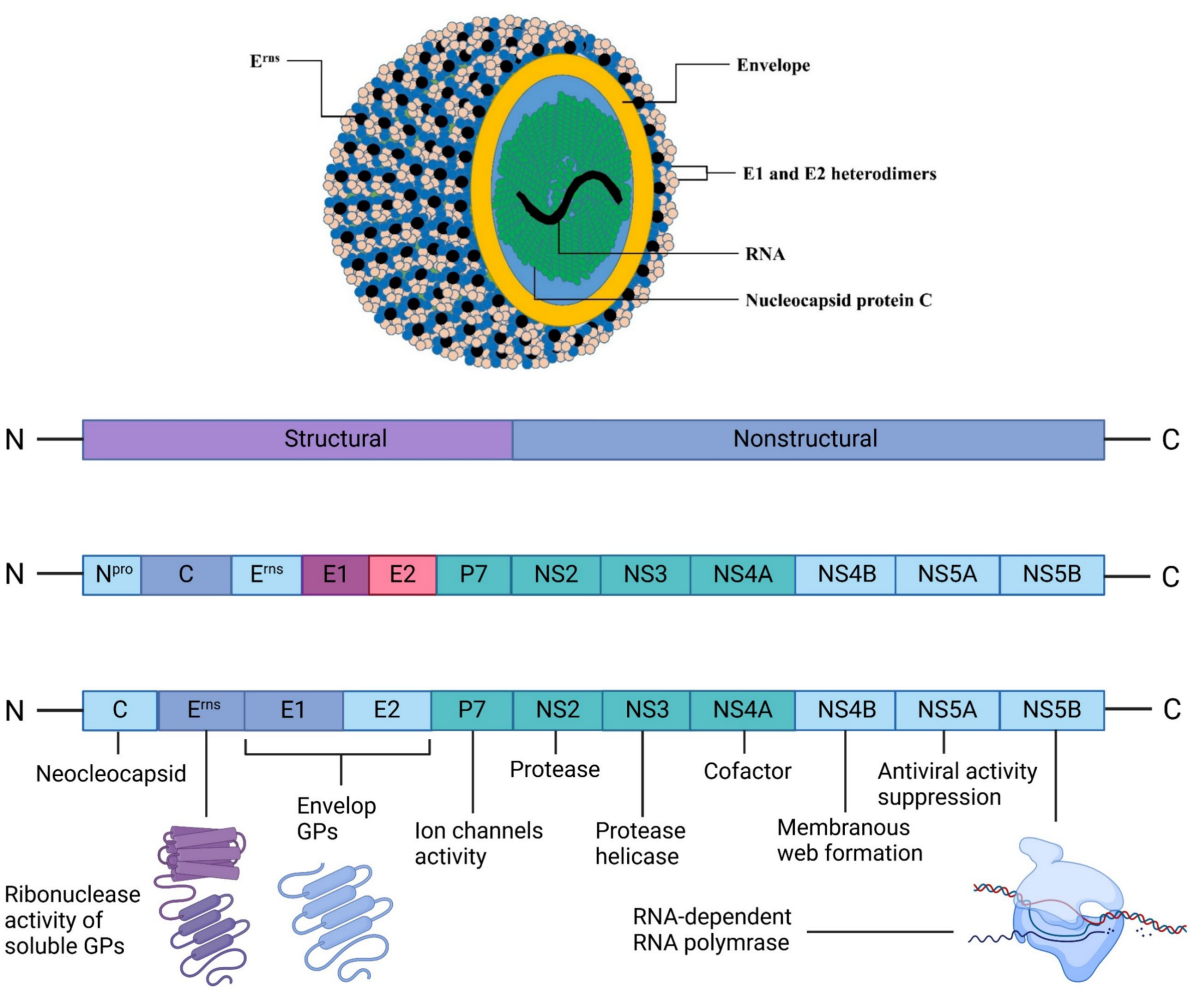
BVDV’s structural morphology and genomic translation into structural and nonstructural proteins for viral assembly. The outermost layer is the lipid envelope, studded with envelope (E) glycoproteins, including the secreted Erns proteins visible as black dots. Beneath the envelope lie the E1 and E2 heterodimers, crucial for viral entry. The yellow layer represents the nucleocapsid protein C, which encapsulates the viral RNA genome (shown in green). Created in BioRender. F. Kulyar, M. (2025) https://BioRender.com/p95yur0.Kulya.

It has been established that cattle are the major source of BVDV infection. Back in 1980, it was the first time isolated from cattle in China, termed Changchun184 and named BVDV1b. Subsequently, ZM-95 (BVDV2) was also isolated in 1995 from pigs, and XJ-04 (BVDV2) and JZ05-1 (BVDV2) were isolated from cattle in China. Until 2013, a complete genomic sequence was also available for pigs for the strains SD0806, ZM-95, and SH-28, indicating 70% similarities (Tao et al., 2013). The structural proteins of BVDV viral particles are Nucleocapsid protein C and glycoproteins, i.e., E1, E2, and E^rns^. These proteins combine with their lipid bilayer, and genomic RNA contains an envelope (Chi et al., 2022). The arrangements of the whole BVDV structure are shown in Figure 1. BVDV genome consists of a single open-reading-frame (ORF) lined with 3′ and 5′ untranslated regions and encodes a polyprotein, which becomes a mature viral protein through undergoing co-translational and post-translational processes. Through phylogenetic analysis (partial sequence) of 5′ untranslated regions of ORF, the N-terminal-autoprotease or envelope-glycoprotein region of BVDV gnome is divided into genetic species, BVDV1 and BVDV2 (Deng et al., 2015).

In a recent study, 53 sequences (5´-UTR nucleotide) were determined using the phylogenetic analysis, and all of them belonged to BVDV1 five subtypes, including BVDV1b, BVDV1c, BVDV1d, BVDV1o, and BVDV1m, with the bootstrap support values of more than 75% for the clade assignments. This strong statistical support offers conclusive proof of the circulation of various subtypes of BVDV1 and elucidates the widespread genomic diversity of the BVDV strains in China typically in cattle in eastern China (Shah et al., 2022; Xiao et al., 2025). The sequence similarities of these subtypes are given in Table 1 (Zhou et al., 2022). Another study revealed 21 BVDV1 subtypes (BVDV1a to BVDV1u), four BVDV2 subtypes (BVDV2a to BVDV2d), and two BVDV3 (HoBi-like) subtypes, which are based on their Brazilian and Thai origins Field (Zhang et al., 2022). However, the most prevalent are BVDV1a, BVDV1b, BVDV1c, BVDV1d, BVDV1u, BVDV1m, BVDV1q, BVDV1p, and BVDV1o, which are continuously circulating in dairy cattle in the whole china (Deng et al., 2020). Further, once the host is infected with BVDV, the noncytopathogenic BVDV has the ability to persistently maintain the infection level before establishing an adaptive immune response by the host (Weng et al., 2015). Additionally, cytopathic-BVDV-strains, the derivatives of non-cytopathic-BVDV-strains, are found due to genetic mutations or cytopathic mutation in persistently infected animals, indicating the higher genetic diversity of BVDV (Wu et al., 2023).

**Table 1:** Sequence similarities of five BVDV1 subtypes.

Subtype	Sequence similarities (%)	Reference strain	Reference
BVDV1c	96.8–99.6	AQMZ02A/21/2	Zhou et al. (2022)
BVDV1d	97.1–98	Lamspringe-735	
BVDV1o	92.2–98	IS25CP01	
BVDV1m	95.1–97.9	ZM-95	

The extent of BVDV infection could be monitored by conducting studies to determine its seroprevalence. Only then, the researchers might implement a control strategy for BVDV. For example, the seroprevalence rate of BVDV in Fujian province for beef cattle is 29.8%, while 92.5% is for dairy cows. Yaks were 53.65 and 72.14% in Tibet and Qinghai provinces, respectively (Yang et al., 2007; Gao et al., 2013). Another study reported BVDV seroprevalence of 14.18, 45.38, 63.27, and 89.49% in water buffaloes, yaks, beef cattle, and dairy cows, respectively (Deng et al., 2015). Alarmingly, even clinically healthy yaks, buffaloes, and cows are 58.09% positive for BVDV antibody, and the BVDV antigen report revealed that around 46.7% of cattle farms were reported positive in China. Overall, a 2.2% rate of persistent infection indicated a considerably higher infection rate in China than other Asian countries (Yang et al., 2023). Several studies have been performed to assess the BVDV infection rate in Xinjiang. For example, an average of 35.40% BVDV prevalence was found in the northern areas of Xinjiang (Yang et al., 2023). Another study conducted a nucleic acid investigation, identified subtype BVDV1b, and found a 39.06% BVDV-positive rate in the Shihezi area of Xinjiang (Li et al., 2009). Yang et al. (2023) conducted an epidemiological investigation in the Kashgar, Aksu, Manas, and Shihezi areas of Xinjiang from 2010 to 2012 and found an 18.61% prevalence rate for BVDV1b. Based on serological investigation, Wang et al. isolated 17 BVDV1 and 4 BVDV2 strains at cattle farms in Xinjiang (Wang et al., 2015). Between 2016 and 2018, Yanhua et al. (2020) found BVDV1q as widespread and prevalent strain at cattle farms of Xinjiang. These reports revealed that BVDV has been spreading in the Xinjiang areas for several previous years, particularly at the cattle farms. In addition, the diverse virus type/subtype poses serious concern on cattle production and causing significant economic losses in animal husbandry in Xinjiang. Thus, by understanding the genetic characterization and prevalence of BVDV could control its dissemination in Xinjiang. Moreover, a comparison among controlled and general BVDV vaccines is made in Table 2.

**Table 2:** Strain wise efficacy comparison of controlled vs. general vaccine.

Factor	Controlled vaccines (modified live virus, killed virus)	General vaccines	References
Homologous vaccine protection	The efficacy for MLV varies 80–95% (viral shedding and clinical signs decreased) inactivated vaccine ranges 60–75%	Its efficacy varies depending upon storage and administration factors	Royan (2009), Fulton et al. (2005)
Heterologous vaccine protection	Univalent MLV (BVDV1) provides partial protection against BVDV2 while multivalent MLV provides cross protection with ≥80% efficacy	According to field data, only partial protection while mismatched strains are main factor of immunity failure	Xue et al. (2011), Palomares et al. (2013)
Protection of fetus	Pre-breeding administration of MLV reduces fetal infection risk up to 90% while inactivated vaccine that of 70%	Lower field efficacy, infections are reported even after immunization	Walz et al. (2017), Stewart et al. (2023)
Cross protection of BVDV3	Comparatively lower efficacy about 10–30%	There are no field reports about its significant protection	Nardelli et al. (2021), Bauermann et al. (2013)
Commercially available vaccine examples	Bovilis^®^ BVDV, Pestigard^®^, Pyramid^®^ 5 + Presponse^®^, CattleMaster^®^ Gold FP^®^ 5, Qilu BVD Inactivated Vaccine, Harbin BVDV-1 MLV Vaccine	Titanium^®^ 5 (MLV), ScourGuard^®^ 4KC (Inactivated), Bovela^®^. CAHIC BVD Inactivated Vaccine, Sinovac BVD-IBR Combination Vaccine	Producers: Merck Animal Health, Zoetis, Qilu Animal Health

## 4 The phylogenetic analysis of BVCV serotypes

**Figure 2:**
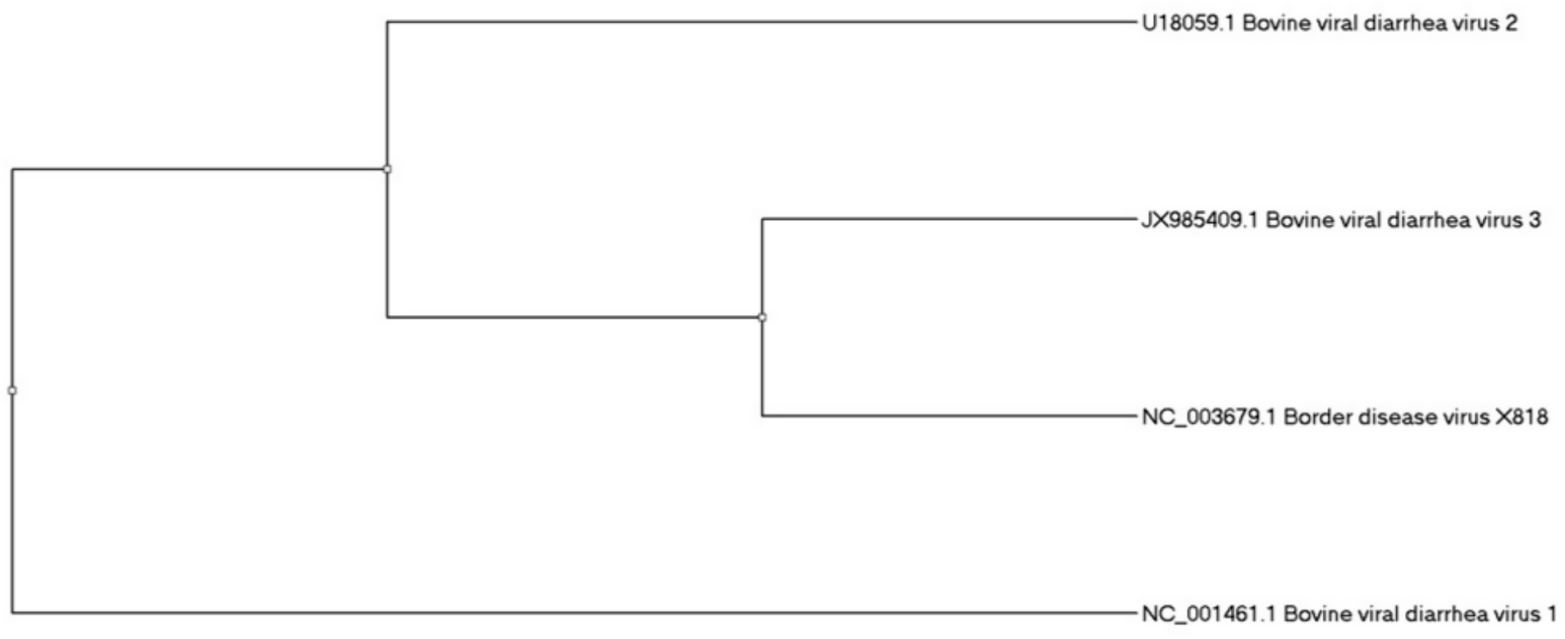
The phylogenetic analysis of *bovine viral diarrhea virus.*

For the understanding of molecular divergence between three BVDV genotypes, we have made a phylogenetic tree (Figure 2). It was made on the base of Npro gene region through Maximum Likelihood method by using reference strains, BVDV1, NADL (NC_001461); BVDV2, 890 (U18059); BVDV3, D32/00_HoBi (JX985409) vs. *Border disease virus* BDV (X818)^1^ as an outgroup. The tree’s proper rooting was enabled by the use of BDV as an outgroup and highlighted the genetic difference among BVDV genotypes (Figure 2).

## 5 BVDV-3 (HoBi-like) vaccine development and constraints in its process

The vaccine development against BVDV-3 (HoBi-like *pestivirus*) starts from the isolation of the virus from infected animals and whole-genome sequencing, which indicated clear genetic distinction from BVDV-1 and BVDV-2, e.g., a polyprotein of about 3,899 amino acids (Yang et al., 2023). Among the targets of the antigens is glycoprotein E2, which plays a role in viral entry and neutralizing antibody induction; recombinant E2 proteins, with truncations to optimize secretion and immunogenicity, have been successful in case of subunit vaccine (Lo et al., 2023). Immunogenicity experiments shows that recombinant E2 could persuade balanced IgG1/IgG2a responses, a sign of strong T cell-dependent immunity, the experimental animal models validating antibody induction and partial protection from fetal infection (Decaro, 2020; Lo et al., 2023). Naturally acquired BVDV-1 and BVDV-2 immunity does not, however, protect against challenge with BVDV-3, highlighting the extent of limited cross-protection and the necessity for BVDV-3–specific or multivalent vaccines for the Xinjiang region, for instance, where more than one genotype is co-circulating. The novel immunizing strategies are being followed for BVDV-3 implication. But antigenic variation, the failure of current vaccines to provide cross-protection, and difficulties in animal model studies hinder progress. Improved diagnostic tests and site-directed vaccine designs must be developed in order to control successfully this newly emerged *pestivirus* (Al-Kubati et al., 2021).

## 6 Assessing the epidemiological patterns of BVDV between Xinjiang and other cattle-producing provinces in China

BVDV is closely related to the loss of productivity in cattle. Assessing which epidemiological factor contribute to this huge loss facilitates the implementation of strategies to reduce the BVDV burden on animals. BVDV initial prevalence, infection risk, intensity of circulation, duration of circulation, testing/culling rate, vaccination, biosecurity, new animals’ introduction to farm, and close contact with the neighboring herds could be measured to investigate the pattern of infection (Pinior et al., 2019). Pinior et al. (2019) calculated €42.14 as an average production loss per animal for BVDV infection. It was demonstrated that higher initial prevalence, infection risk, intensity of circulation, and duration of circulation could significantly increase the average production loss per animal to €67.19, indicating that introducing a new batch of cattle into the healthy cattle herd is the most vital factor that facilitates BVDV transmission. Several analyses revealed that purchasing pregnant cows considerably increases the BVDV infection rate in the healthy cattle herd (Bitsch et al., 2000; Santman-Berends et al., 2017; Pinior et al., 2019). Further, interaction/contact with neighboring animals or cattle herds or contact with the housing of other ruminants is a critical factor that boosts BVDV viral transmission from the infected to the healthy herd and, therefore, leads to production losses. If farmers permitted their cattle to be in contact with other cattle of any other herd or introduced new animals into their farms could substantially contribute to 18% average production losses per animal. Alarmingly, the average production losses per animal are twice as high in dairy herds compared with beef herds annually (Graham et al., 2016; Kaiser et al., 2016; Pinior et al., 2019). Further, average production losses per animal could be 28–29% lower using the biosecurity measures and 8–12% lower by following proper vaccination schedules. Moreover, if the cattle herd is vaccinated with the BVDV vaccine, the fetal infection proportion could be 85% reduced, and the abortion rate could be 45% lower. The reasons farm owners face huge economic losses are either because the farmers fail to follow the vaccine schedule properly or because they administer unprotected vaccines whose efficiency has not been verified. Some studies described that testing/culling of the animals is not a major factor that could change average production losses per animal because it could not be considered an economic strategy (Newcomer et al., 2015; Evans et al., 2019; Pinior et al., 2019). Yaks are of significant importance in China’s cattle industry. The BVDV prevalence, a main epidemiological factor, is found higher (67.5%) in yaks in Xinjiang compared with its prevalence in Qinghai and Sichuan that is 35.5 and 31.16%, respectively, indicating a significant impact of BVDV infection on yaks in Xinjiang (Diao et al., 2020). Another study collected animal serum and found a positive rate of 37.5% in Xinjiang, which is still higher than in Tibet (10.77%), Ningxia (30%), and Shaanxi (4.57%) (Chang et al., 2021). Zhang et al. (2022) reported that in Xinjiang, BVDV infection was significantly spreading (99.33%) in the cattle farms of non-BVDV-vaccinated animals. As the chance of BVDV infection spreading rate increases by importing dairy cattle from Australia to Xinjiang (Zhong et al., 2011), repeated circulation of dairy animals at domestic levels further boosts cross-species BVDV infection (Weng et al., 2015). Lang et al. (2014) identified nine BVDV sub-genotypes that were already circulating in dairy animals in China. These reports exhibit that assessing epidemiological patterns of BVDV infection in Xinjiang could help manage the BVDV infection rate. Moreover, some studies indicating BVDV’s prevalence via different methods are summarized (Table 3).

**Table 3:** The BVDV’s prevalence in Xinjiang and some other cattle producing provinces.

Province	Prevalence of BVD (%)	Description	References
Xinjiang	35.40	Average prevalence: mainly BVDV-1 type.	Yang et al. (2023)
Xinjiang	39.06	The nucleic acid positive rate in Shihezi region.	Zhang et al. (2022)
Xinjiang	18.61	Multiple areas average prevalence typically prevalent strain BVDV-1b.	Manandhar et al. (2018)
Xinjiang	53.68	The positive rate by antibodies detection varies in region wise.	Yang et al. (2023)
Gansu	93.33	Multiple strains were detected higher in large dairy farms.	Zhang et al. (2022)
Ningxia	93.33	Higher infection rates as in Gansu province.	
Inner Mongolia, Shaanxi, Tibet and Qingai	93.33	Significant infection rates were found in different regions	

## 7 An insight into pathogenic mechanisms of BVDV

**Figure 3:**
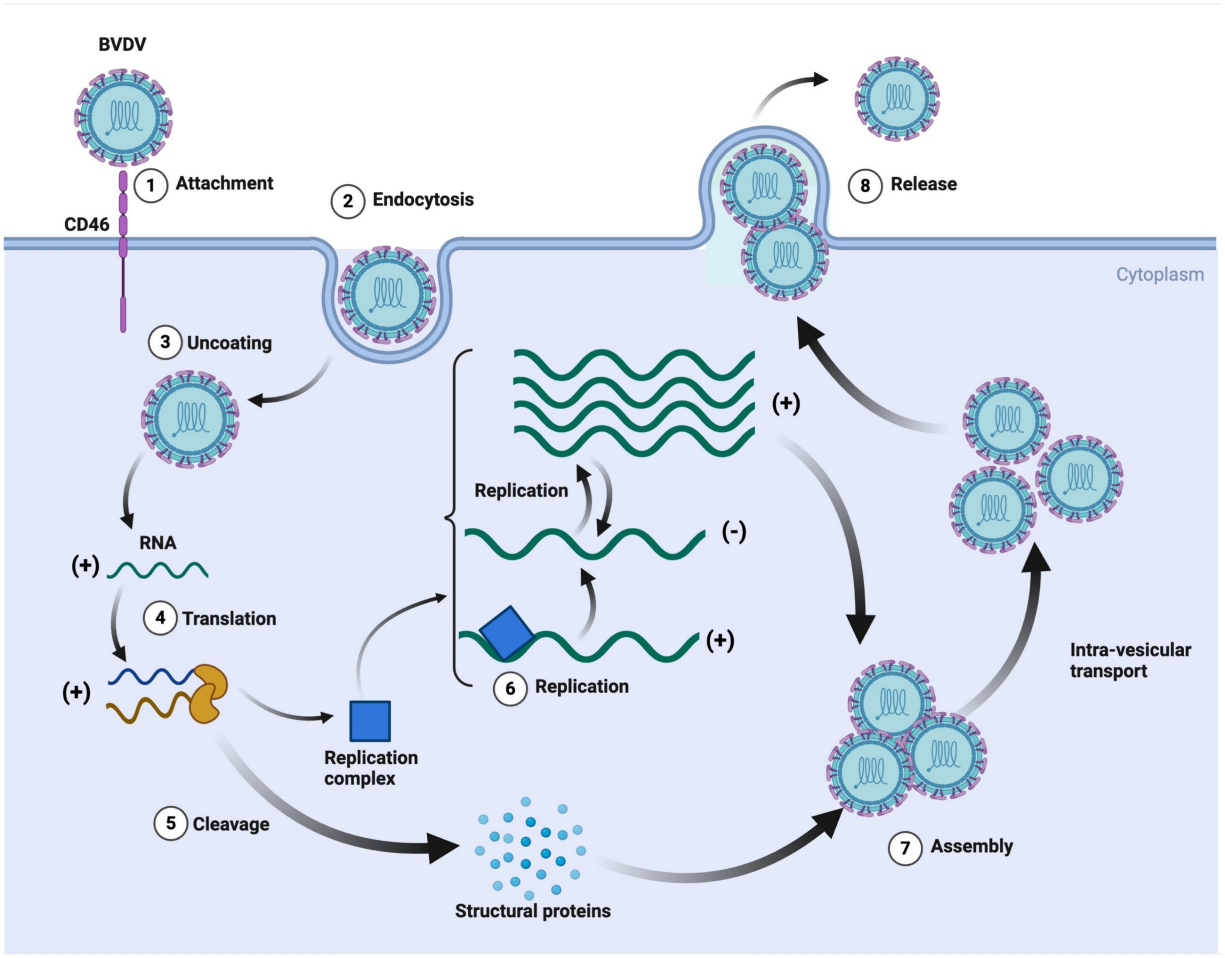
The replication cycle of bovine viral diarrhea virus (BVDV). This detailed schematic illustrates the key steps in BVDV infection and replication. The process also highlights the formation of the replication complex and the production of structural proteins, emphasizing the intricate cellular mechanisms exploited by BVDV for its propagation. It further provides insights into potential targets for antiviral strategies and vaccine development against BVDV. Created in BioRender. F. Kulyar, M. (2025) https://BioRender.com/6penelu.

BVDV pathogenic mechanism is highly dynamic, including initial entry, attachment, replication, assembly/release, and spreading of the infection (Qi et al., 2022). The whole process is very complex, and the mechanism is still unknown. However, a few studies explain its mechanism (Rajput et al., 2017; Ma et al., 2022). BVDV starts its life cycle after attachment with the host cell, following endocytosis through binding with the host cell receptor. BVDV proteins, i.e., E^rns^, E1, and E2, help in binding and endocytosis. BVDV RNA constructs its replication complex and structural proteins, which help in BVDV viral assembly and, finally, its release from the host cell (Ma et al., 2022). The possible mechanism is shown in Figure 3.

**Figure 4:**
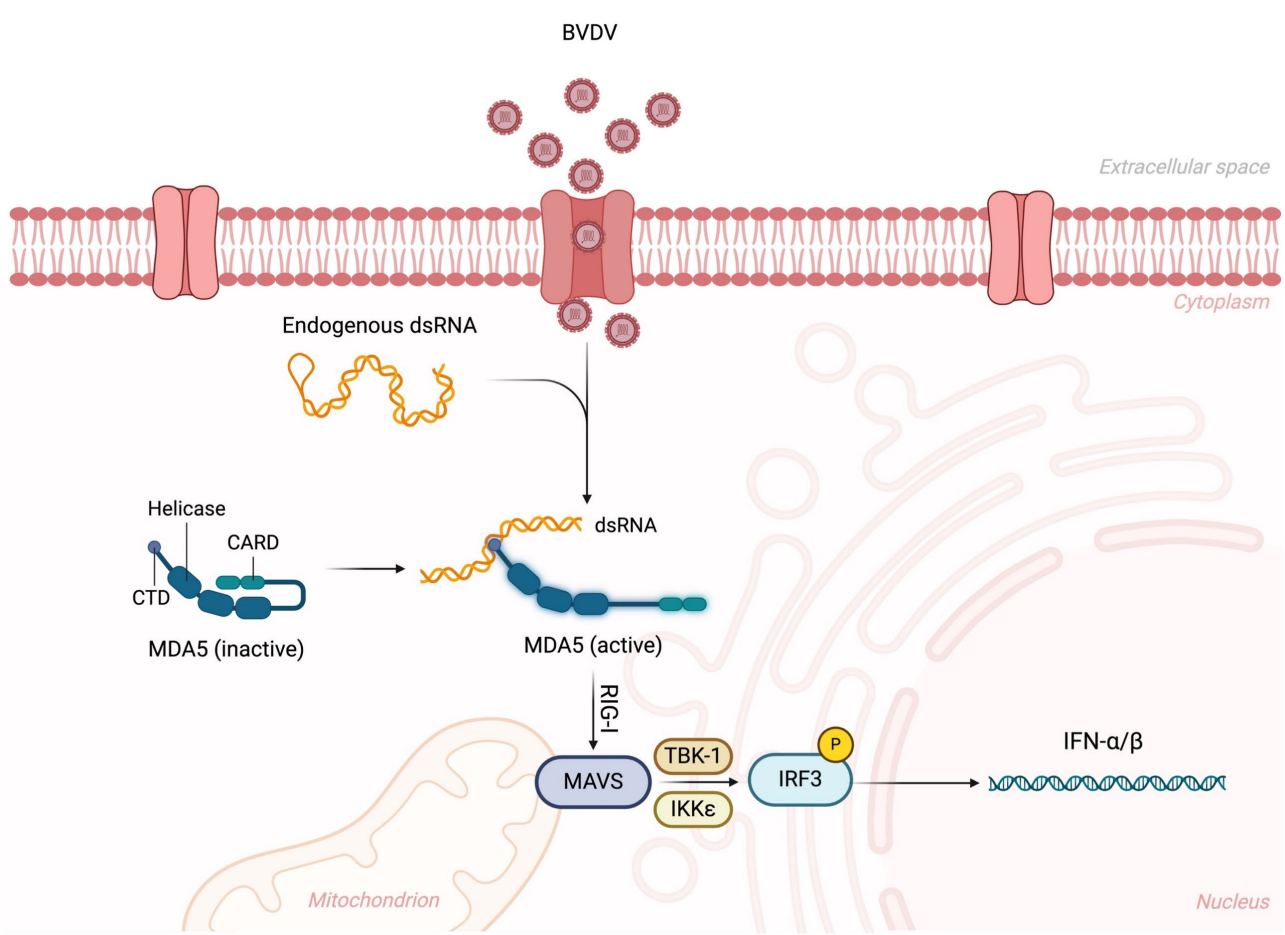
BVDV-mediated inhibition of RLR signaling pathway. The virus is recognized by the RLR sensor MDA5 (melanoma differentiation-associated protein 5), leading to its activation. This activation triggers the downstream signaling cascade, where MDA5 interacts with the mitochondrial antiviral-signaling protein (MAVS), leading to the recruitment of TBK1 and IKKε kinases. These kinases phosphorylate IRF3 (interferon regulatory factor 3), promoting its translocation to the nucleus and the subsequent production of type I interferons (IFN-α/β). Created in BioRender. F. Kulyar, M. (2025) https://BioRender.com/glqkilx.

BVDV virus has a complex interaction with the hosts for entering their body and attachment/entry into their cells, which is performed through different affinities and extremely active processes. Before the cellular entry into host cells, the BVDV virus must establish close contact with the host’s cells after traveling to the suitable site. For this purpose, the BVDV virus performs diffusive motion and confined or directed motions (Riedel et al., 2020). It has been found that the BVDV virus requires a receptor (bovine CD46) to enter into host cells. CD46 is ubiquitously expressed, a cofactor, and involved in autophagy modulation and T cell regulation. Some scientists encoded BVDV with mCherry-E2-fusion-protein (BVDV E2) and investigated the attachment/entry mechanism. BVDV E2 binds with CD46 through 30 C-terminal amino acids of complement control protein module-1 (Krey et al., 2006; Riedel et al., 2020). BVDV entry is immediately supported with viral replication and its transmission. Strong et al. (2015) performed strand-specific RT-PCR and demonstrated that the BVDV virus was actively replicating in the blood samples of animals even 85 days after infection, which is crucial and indicates how BVDV persistently infects cattle herds. BVDV virus also has the ability to escape from the host’s immune system, which increases BVDV’s persistent infection rate and inhibits the host’s innate immune response. It has been found that BVDV inhibited the IFN-β production and RLR-signalling pathway by interacting NS4B with 2CARD of the host’s MDA5 domain, resulting in increased BVDV1a proliferation. RLR is the RNA sensor in the cytosol, and RIG-I, MDA5, and LGP2 are its three major members. RLR contains a central helicase domain and CTD, which together play roles in the detection of immunostimulatory RNAs (Rehwinkel and Gack, 2020; Chi et al., 2022). BVDV-mediated inhibition of the RLR signaling pathway is shown in Figure 4.

Based on clinical manifestations, in a study of 177 positive samples of BVDV, 30 and 70% of isolates from cattle were cytopathic and non-cytopathic, respectively. It was also crucial that almost 72% of isolates were obtained from cattle below 1 year of age, and 34% of isolates were recovered from the calves (4–6 months). The clinical signs were 18% for diarrhea, 19% for abortion, and 27% for respiratory syndrome, indicating that BVDV virulence might be affected by several factors (Ahn et al., 2005). BVDV virus caused 33 to 88% and 4 to 8% morbidity and mortality, respectively, and BVDV infection could be divided into four categories, i.e., mild acute, severe acute, chronic, and mucosal disease (Ridpath et al., 2006). BVDV viral antigen was found under the tonsil’s lymphoid tissue, indicating that the tonsil epithelial crypt could be the possible entry site of the non-cytopathic BVDV virus (Liebler-Tenorio et al., 2002). Another study determined that BVDVq1 could increase the size of mesenteric-lymph nodes and cause intestinal and renal hemorrhages and intestinal inflammation in calves in Xinjiang (Yanhua et al., 2020). It has also been found that inoculation of BVDV2 induced acute BVDV infection in calves with symptoms of diarrhea, depression, and fever and also caused recumbency in calves because it significantly decreased lymphocytes and thrombocytes. Other BVDV isolates could also cause severe hemorrhagic diathesis (Liebler-Tenorio et al., 2002). Additionally, Seong et al. (2013) investigated the effects of Korean non-cytopathic BVDV1 and BVDV2 in calves and compared the pathogenetic differences between these isolates that are given in Table 4. Any animal in the cattle herd is susceptible to BVDV infection through horizontal transmission. After exposure, the BVDV incubation period is 6–12 days, but every BVDV strain has some variation in the incubation period, which depends upon the dose of the transmitted virus. Post BVDV infection, the infected animal starts shedding the virus between 3 and 15 days, with a maximum of 3 weeks (Evans et al., 2019). Previous studies indicate that the genotypes of BVDV have different antagonizing abilities with host’s immune system. For example, BVDV1a could potentially overwhelm IFN-β production via IRF-3 as compared to BVDV2a. While, evasion mechanism of BVDV3 remains less prominent. Since there exists sequence difference in immune-modulating proteins like Npro and NS4B among genotypes, molecular docking and in silico modeling of host-pathogen interactions (e.g., Npro–IRF3, NS4B–MDA5) can provide predictive information on genotype-specific immune evasion and rational vaccine development against BVDV3 (Pang et al., 2023; Wang et al., 2024).

**Table 4:** Pathogenetic differences between non-cytopathic BVDV1 and BVDV2 in calves (Seong et al., 2013).

Pathogenetic factors		Non-cytopathic BVDV1				Non-cytopathic BVDV2			
		3 h	3 dpi	6 dpi	9 dpi	3 h	3 dpi	6 dpi	9 dpi
Clinical signs	Respiratory distress	–	Mild	Moderate	Mild	–	Mild	Severe	Severe
Leukocytes	Leukopenia	Mild	Mild	Mild	Mild to moderate	Mild	Mild to moderate	Severe	Moderate
Platelet count		–	Decreased	–	Increased	Decreased	Increased	Severely decreased	–
Lymphocytes		–	Decreased	Further decreased	Increased	–	Decreased	Further decreased	Increased
Monocytes		–	–	Increased	Decreased	–	–	Increased	Increased
Apoptosis		Mild	Severe	Mild to severe	Mild	Severe	Severe	Severe	Mild
Proinflammatory cytokines (pg/mL)	TNF-α	–	–	Decreased	Increased	–	–	Increased	Decreased
	IFN-γ	Increased	Persistent	Severely increased	Decreased	Increased	Decreased	–	Decreased

h, hours; dpi, days post-infection; (−) no symptoms.

## 8 BVDV-induced immunosuppression and autophagy significantly contribute to secondary infections in the cattle of Xinjiang

**Figure 5:**
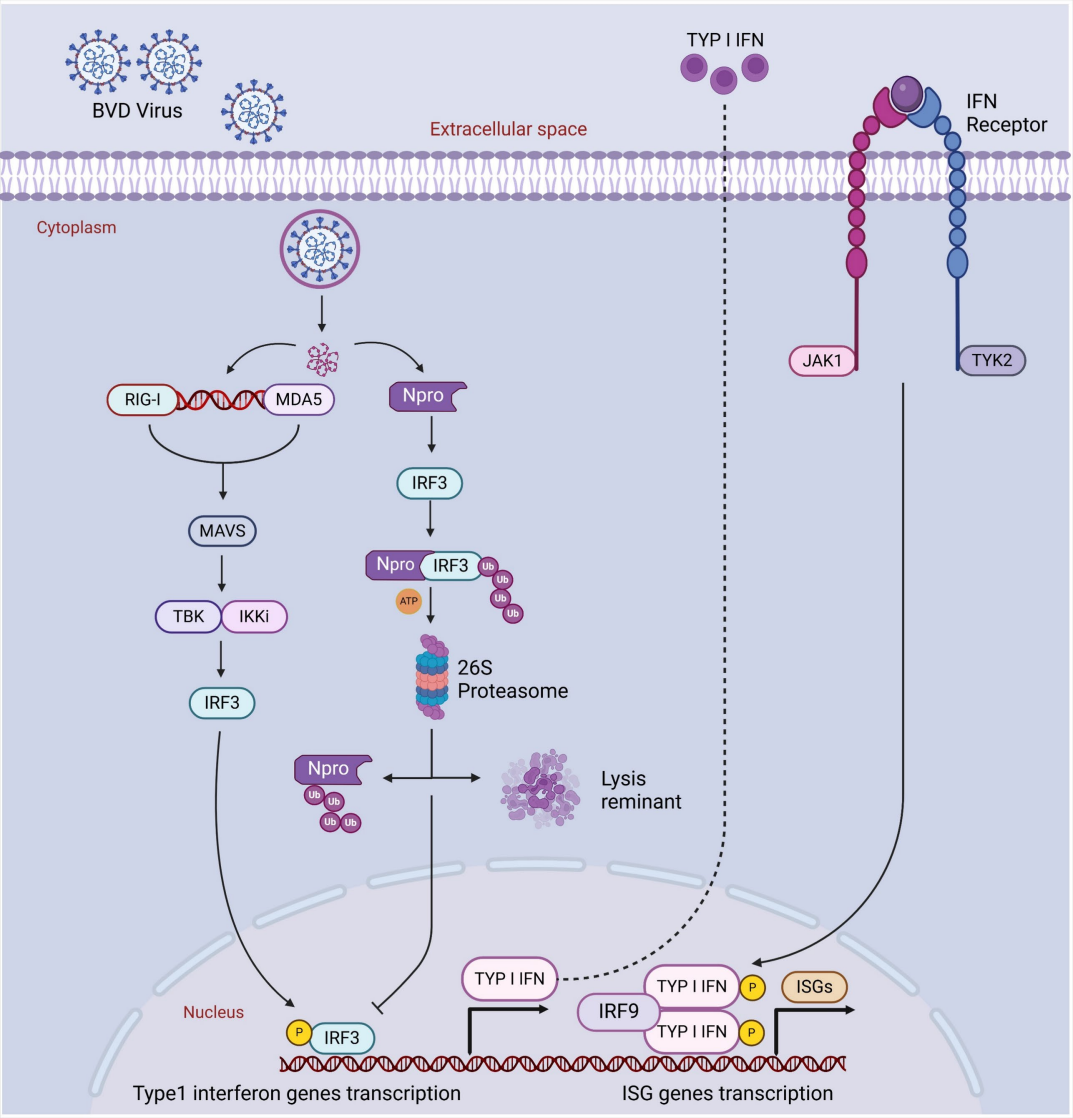
The blockage of host’s IFN activated immune response by Npro and IRF3 degradation. Cellular PRRs recognize PAMPs of viral infection. Such recognition thus triggers a sequence of cellular pathways that eventually lead to the translocation of phosphorylated IRF3 into the nucleus and commence the transcription of type I interferon genes through binding to IFN-α/β promoters. Npro might bind with IRF3 prior to its activation induced by phosphorylation, thereby targeting IRF-3 for ubiquitination and subsequent proteasomal degradation, resulting in a block in the type I interferon response. Created in BioRender. F. Kulyar, M. (2025) https://BioRender.com/j4sbip1.

It has been reported that BVDV has a higher proportion of inducing mixed infections with other pathogens in the host. BVDV infection suppresses the host immune response, resulting in immunosuppression and an infected animal promotes secondary infections through already present pathogenic organisms in the host. Recently, BVDV-induced secondary infections have become a common issue in Xinjiang. With these mixed infections, *Pasteurella multocida* and *Mycoplasma* routinely cause a higher mortality rate in calves and cattle, weakening host immune response in the animals of Xinjiang (Zhang et al., 2022). It has been found that the BVDV virus suppresses the response of IFN α/β of peripheral blood mononuclear cells (monocytes, natural killer, B, and T cells) and decreases T cells (CD4^+^ and CD8^+^) and lymphocytes levels in blood circulation, contributing to systemic immunosuppression in the persistently-infected animals (Weng et al., 2015; Liu et al., 2023). Compared with the control group, BVDV infection significantly decreases CD3^+^ (55.8%), CD4^+^ (53%), and CD8^+^ (56.7%) cells in the BVDV-infected group (Liu et al., 2023). BVDV evasion capability maintains persistent infection levels by disrupting adaptive immune response (Weng et al., 2015). In persistently infected animals, BVDV is triggered in early fetal development, and the BVD virus prepares to successfully evade the host’s innate immune response by inhibiting IFN α/β expression. Although some studies explain the BVDV evasion mechanism, its exact details still need to be clarified. For example, IRF-7 and IRF-3 are the main transcriptional factors that regulate type-I-IFN. At first, E^rns^, a BVDV structural protein, inhibits type-I-IFN through degrading dsRNA (virus). Secondly, N^pro^, a non-structural BVDV protein, ceases IFN α/β expression through targeting IRF-3. Usually, N^pro^ triggers IRF-3 proteasomal degradation and polyubiquitination and induces its long-term downregulation. BVDV decreases IRF-7 and IRF-3 expression levels in persistently infected cattle, indicating how BVDV disrupts and evades the host’s innate immune response and significantly contributes to host immunosuppression (Figure 5) (Platanias, 2005; Schmid et al., 2010; Weng et al., 2015).

Another study revealed that E^rns^ of BVDV significantly inhibits lymphocyte protein synthesis but avoids damage in their cell membranes, indicating that BVDV could trigger apoptosis, leading to autophagy (Fu et al., 2014). Fu et al. also described that BVDV infection increased autolysosome, the autophagosome, and ATG14 and Beclin1 expression, suggesting BVDV significantly induced autophagy (Fu et al., 2014). Although autophagy is an important mechanism to minimize the virulence effects of viruses in the hosts, BVDV induces autophagy in host cells to promote its replication process and to suppress the host’s innate immune response (Yang et al., 2022). Further, BVDV also causes severe clinical diseases in wild animals along with cattle and other domestic animals. It may include malformations, mucosal disease, abortions, thrombocytopenia, and reproductive disorders. In acute BVDV infection, a severe inflammatory response has been initiated in the gastrointestinal tract (Li et al., 2019). Thus, immunosuppression, along with non-specified clinical symptoms in the BVDV-infected animals, is also endangering other healthy animals in Xinjiang (Diao et al., 2020; Su et al., 2022). Therefore, corresponding preventive plans are necessary to control BVDV infection dissemination in Xinjiang.

## 9 Preventive strategies in controlling the spread of BVDV across China

Preventive and control strategies for BVDV are critical in the cattle industry. Although vaccination is a significant measure in controlling BVDV infection, culling the persistently infected cattle could also be adopted. Compared to the European countries, China has not launched any BVDV eradication program (Yang et al., 2023). Currently, the BVDV vaccine developed from conventional cell lines belongs to BVDV1 and BVDV2 strains. Both inactivated and modified-live vaccines (MLV) are available for use against BVDV infection around the globe (Evans et al., 2019). However, the vaccine against BVDV3 (HoBi-like virus) is not commercially available yet, and this virus is also affecting buffaloes, goats, and sheep as well (Kalaiyarasu et al., 2023). BVDV vaccine is helpful in the prevention of developing persistently infected animals, protecting fetuses from infection, and preventing transient infections. Vaccination also boosts the growth rate and regulates the immune responses of the calves exposed to the persistently infected animals, indicating that BVDV-associated production/reproduction losses could be managed using a proper vaccination schedule (Grooms et al., 2014; Evans et al., 2019).

Controlled and coordinated eradication campaigns have been conducted successfully in the European countries. The campaigns aim primarily at the identification and elimination of persistently infected (PI) animals, which are the primary reservoir of BVDV maintenance and transmission in cattle herds (Lindberg et al., 2006). The Scandinavian countries like Norway, started a nationwide voluntary BVDV control program 1992, than to compulsory nation eradication scheme 2005, through serological screening and culling of PI animals as well as the movement restriction (Valle et al., 2005), as a result Norway declared as BVDV free country 2018 (Toplak et al., 2021). Sweden also achieved BVDV free status through similar strategies with a difference, they followed bulk milk antibody testing for identification (Ståhl et al., 2002).

**Figure 6:**
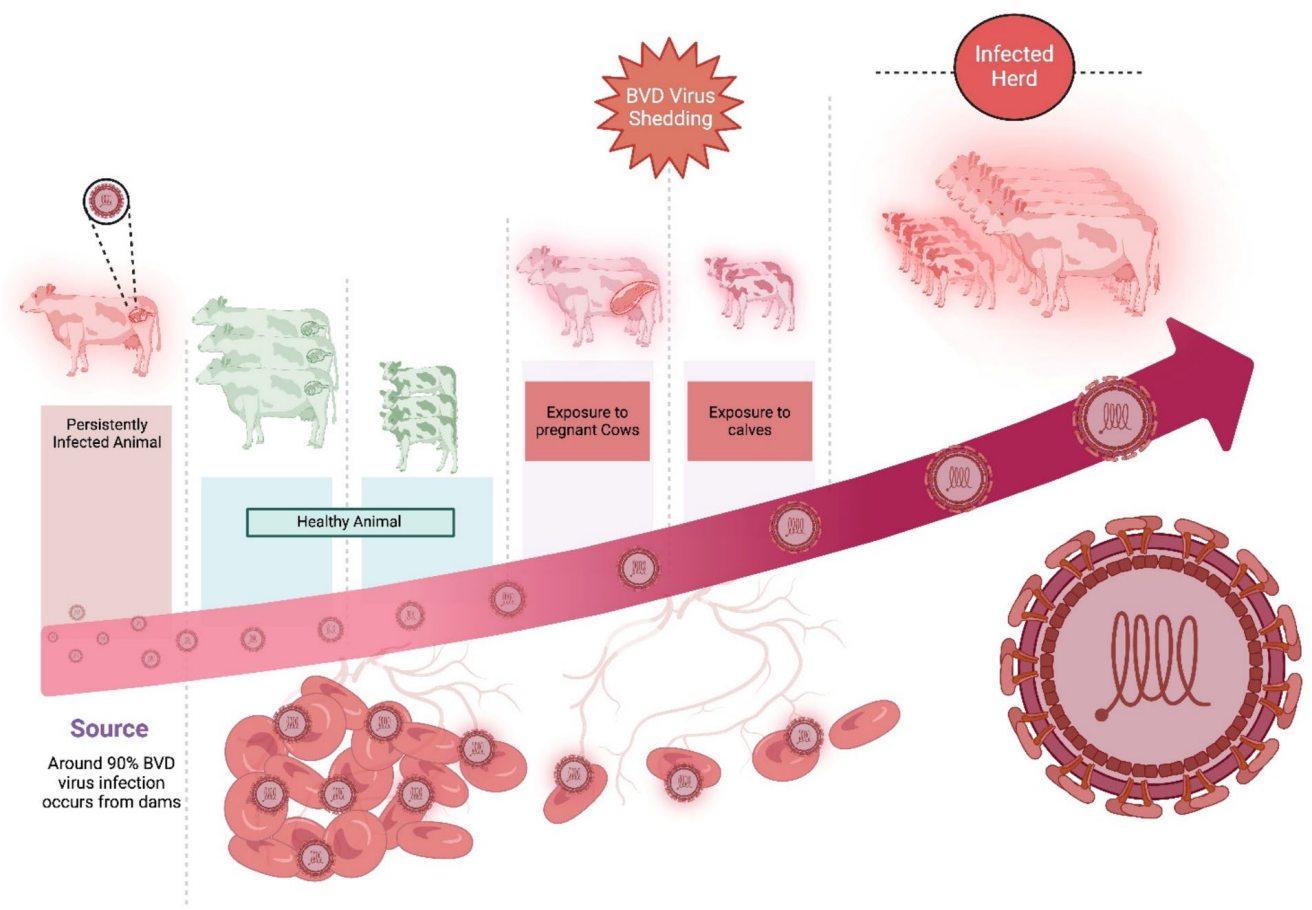
The dissemination of BVDV from persistently infected animals to other healthy animals and calves. Created in BioRender. F. Kulyar, M. (2025) https://BioRender.com/m5lufia.

As BVDV has the potential to spread across borders, bovine viral diarrhea (BVD) is enlisted as a notifiable infection in the Office International des Es (OIE) list. Every country has certain principles which are strictly followed to restrict those animal trading that has an unknown bovine viral diarrhea status (Marschik et al., 2018). Preventing the development of new persistently infected animals and reducing the prevalence of these animals in any cattle herd are the fundamental principles in controlling and eradicating BVD. It includes (a) testing (identifying) and culling (removing) of the persistently-infected animals, (b) improving biosecurity (reducing virus transmission in the population), and (c) vaccination (protecting the fetus from BVDV infection), which reduces the development of persistently-infected animals. Further, strictly controlling the semen quality and embryo transferring protocols could also control BVD at the farm level by following the closed-herd policy. Upon failing the closed-herd-policy, direct or indirect BVDV transmission routes are controlled. For this, purchasing persistently-infected animals and introducing dams containing persistently-infected fetuses are immediately stopped. Further, putting a double fence on the boundary wall, keeping recently purchased animals in quarantine, and cleaning the shared equipment/vehicles between the herds were strictly followed (Evans et al., 2019; Benavides et al., 2021; Schweizer et al., 2021). The dissemination of the BVDV virus shed from BVDV-infected animals is shown in Figure 6.

The application of control strategies for BVDV in Xinjiang and rural China in general is hindered by a number of factors. Cattle production in Xinjiang is largely extensive or semi-nomadic in nature, and fencing and separation of the animals is not feasible. Furthermore, poor farmer education on viral spread, lack of accessibility of veterinary diagnostics, and uncontrolled inter-farm cattle movement significantly decrease the efficacy of such control measures. Focused education campaigns and government-funded infrastructural development are desperately necessary to enable the institution of these protocols in rural and pastoral communities. Moreover, inadequate veterinary extension services and limited knowledge regarding PI animal recognition prevents early detection and culling. Such systemic limitations lead to the failure of compliance with vaccination schedules, especially in remote counties like Hami and Manas. In the absence of increasing farmer education, access to diagnostic infrastructure, and affordability of vaccine delivery, regional control programs will remain inadequate despite apparent epidemiologic justification.

Unfortunately, non-systematic vaccine approaches have also been extensively applied and whether these approaches substantially reduce the infection rate is unknown. Limited data regarding such vaccination approaches indicates several issues in the recently available vaccines. For example, it is unknown whether fetal protection is 100% or not because the BVDV virus crosses the placenta, resulting in reproductive and fetal health issues. To protect the fetus from BVDV infection, either cell-mediated immunity or antibody neutralization is useful, but it is also not clear (Evans et al., 2019). Thus, further investigations are still required on how dam or fetus health could be protected from BVDV infection.

## 10 Conclusion

BVD is enlisted as a notifiable infection in the Office International des Epizootics (OIE) list because BVDV has the potential to spread across borders. BVDV infection has appeared as a serious threat to the cattle industry, particularly in Xinjiang, China. It infects cows, buffaloes, pigs, goats, yaks, and sheep and is contributing to the huge economic losses in this industry. BVD virus has dynamic genetic characteristics, and rapidly discovering new biotypes are due to cytopathic mutation in persistently infected animals, indicating the interconversion of non-cytopathic to cytopathic. This nature of BVDV increases the infection rate in Xinjiang compared with its prevalence in Beijing, Fujian, Shaanxi, and Shandong. The pattern of BVDV infection depends upon the infection risk, intensity/duration of circulation, testing/culling rate, arrival of new animals, and close contact with the neighboring herds. Thus, identifying and removing the persistently infected animals and improving biosecurity, i.e., reducing virus transmission in the population, could minimize the BVDV infection rate. Although vaccination is a useful tool to prevent the BVDV infection rate, applying non-systematic vaccine approaches should strictly be avoided. Further, the BVDV virus’s diffusive, confined, and directed motions facilitate its replication and transmission. Its active replication in the blood samples of BVDV-infected animals, even 85 days after infection, indicates virulence of BVDV persistently infects cattle herds, which increases morbidity and mortality rates. Additionally, immunosuppression and autophagy, along with non-specified clinical symptoms in the BVDV-infected animals, also endanger other healthy animals in Xinjiang.

Attention must be paid to the preventive measures of BVDV infection. Preventing the development of new persistently-infected animals, controlling the semen quality, maintaining the embryo transferring protocols, following closed-herd-policy, prohibiting the introduction of dams containing persistently infected fetuses, putting double-fence on the boundary wall, keeping recently purchased animals in quarantine, and cleaning the shared equipment/vehicles between the herds should be strictly followed. Xinjiang should be particularly focused because its major districts Kuitun, Shawan, Yili, Manas, Aksu, Hami, and Shihezi contains 4 million cattle, it borders with the Kazakhstan, Central Asian countries, and Russia, and most of the Holstein cattle in Xinjiang were often imported from Australia where BVDV1c is reported a predominant subtype. It is predicted that the above factors could increase the BVDV infection rate in the healthy herd of cattle in Xinjiang. Thus, the risk of BVDV infection could be minimized by decreasing the prevalence rate, understanding its epidemiology and pathogenesis, and using approved and verified BVDV vaccines in cattle.

## Footnotes

^1^ www.ncbi.nlm.nih.gov
